# Facilitators and barriers to using physical activity smartphone apps among Chinese patients with chronic diseases

**DOI:** 10.1186/s12911-017-0446-0

**Published:** 2017-04-19

**Authors:** Liu Sun, Yanling Wang, Brian Greene, Qian Xiao, Chen Jiao, Meihua Ji, Ying Wu

**Affiliations:** 1 School of Nursing, Capital Medical University, 10 Xitoutiao, Youanmen, Fengtai District, Beijing, China; 2 School of Nursing, University of Pittsburgh, Pittsburgh, PA USA

**Keywords:** Chronic disease, Physical activity, mHealth, Exercise, Health informatics

## Abstract

**Background:**

Smartphones and their applications (apps) impact society and health care. With the growth of smartphone users and app downloads in China, patients with chronic diseases have access to a self-management strategy for physical activity. Although studies report physical activity apps improve the physical activity of patients, data is limited concerning their use of these apps. Therefore, this study investigated the current usage, willingness to use, and barriers to using physical activity apps of Chinese patients with chronic diseases.

**Methods:**

We designed a questionnaire to collect data from chronic disease patients in a tertiary hospital in Beijing, which was sent to 250 patients in four departments.

**Results:**

Two hundred eighteen questionnaires were returned (87.2% response rate). Most (92.7%) respondents owned a smartphone, 34.9% had used a physical activity app, and 18.8% were current users. Additionally, 53.7% were willing to use a physical activity app designed for them. Respondents more likely to use physical activity apps were younger (i.e., ≤ 44 years), more educated, current smartphone users, and previous users of physical activity apps; moreover, they believed they needed exercise, their disease required exercise instruction and support, and their physical status needed monitored when exercising (*p* < 0.05). Main barriers to using apps reported were insufficient function, difficulty of use, extra cost, and security issues.

**Conclusions:**

Our results indicate sizeable smartphone ownership among Chinese patients with chronic diseases; moreover, over half of our participants report they would use a physical activity app designed for them. This information can be leveraged by healthcare workers managing patients with chronic diseases.

**Electronic supplementary material:**

The online version of this article (doi:10.1186/s12911-017-0446-0) contains supplementary material, which is available to authorized users.

## Background

As of July 2012, about 260 million Chinese have been diagnosed with chronic diseases (e.g. Cardiovascular Diseases, Respiratory Diseases and Diabetes Mellitus) that are responsible for 85% of the total deaths and 70% of the total medical cost in China [1]. These chronic diseases are a growing public health concern in not only China, but also Western countries. Mortality can be reduced and quality of life improved by reducing health behaviors related to risk factors. Physical inactivity is modifiable risk factor that contributes to the premature death of patients with cardiovascular disease, cancer, diabetes mellitus, and other chronic diseases [2]. Although (1) physical activity is associated with improved physical function, psychological well-being, mortality rates [3, 4], and overall health, and (2) physical activity can be made inexpensive, accessible, and acceptable to patients, the percentage of Western patients who reach exercise goals remains suboptimal [5]. For example, 85% of Canadian adults and 97% of American adults fail to meet public health guidelines for physical activity [6]. In response, attempts are being made to promote physical activity through the use of smartphone apps. The same situation is occurring in China [7].

Smartphone and mobile technology have become ubiquitous. As of July 2015, the China Internet Network Information Center reports that 594 million Chinese are cellphone netizens (i.e., active users of the Internet via Internet-enabled devices) [8]. In addition, a number of mobile health (mHealth) technology advances (i.e., health-related smartphone apps) are improving lifestyles and altering care delivery. In 2012, the number of health-related smartphone apps available on the Mac App Store was over 13,000 and rising, of which 6.97% were exercise related [9]. In May 2013, iTunes and Google Play offered 875,683 and 696,527 active apps, respectively, and 41,246 of these were categorized as *health and fitness* [10]. Knight et al. searched iTunes and Google Play mobile app stores in 2014 and found 2,400 physical activity apps, of which 379 were eligible in English [6]. Our previous study analyzed the iOS App Store and the four main Android app stores in mainland China, and we found 310 iOS and 220–400 Android physical activity apps were available, and 16.3–44.3% of them had been downloaded over 10,000 times [11].

Research about the use of smartphone apps for improving physical activity is in its early stages, but increasing. For example, Fanning et al. conducted a meta-analysis on increasing physical activities with mobile devices and concluded that it was an effective means for influencing physical activity behavior [12]. In a systematic review, Stephens et al. assert that smartphone apps and text messaging interventions are indeed innovative and well-accepted strategies to reduce specific cardiovascular risk factors (e.g., decreased weight, waist circumference, and body mass index) [13]. More recently, in a report of the SMART Move randomized controlled trial to evaluate the effectiveness of a smartphone app in increasing physical activities in primary care, Glynn et al. demonstrate that a self-designed app increased physical activity over 8 weeks in a primary care population [2]. In addition, in a review of apps for promoting physical activity that included 15 eligible studies (i.e., six were qualitative research studies, eight were randomized control trials, and one was a non-randomized study with a pre-post design), Steven et al. assert apps can be effective in promoting physical activity [14]. In a more recent systematic review that examined the efficacy of 21 interventions that use apps to improve diet, physical activity, and sedentary behavior in children and adults, Schoeppe et al. [15] present 14 studies that targeted physical and provided statistically significant health improvements. All of these studies not only provide evidence that app-based interventions to improve physical activity can be effective, but also suggest that apps may become an important driver for promoting physical activity.

However, the application of these apps requires an adequate understanding of the disposition of patients to accept and adhere to interventions that feature the apps. Moreover, although considerable research on this topic has been conducted in the Western context, the collection of data vis-à-vis the smartphone app use of the Chinese population is only beginning. As a result, we have a limited understanding of the user preferences of the Chinese population concerning smartphone apps—espeicially ones related to health. It is against this backdrop that we sought to examine (1) the dispositions and willingness of Chinese chronic disease patients to using a physical activity app and (2) the characteristics of patients who likely to use physical activity apps (3) the barriers these patients reported to using the apps. Our results can inform subsequent research to integrate smartphone physical activity apps into interventions to engage patients with chronic diseases—in either a Western or Chinese context—in the self-management of their physical activity behaviors.

## Methods

### Setting

This study was conducted in four outpatient departments (i.e., cardiovascular, respiratory, endocrinology, and cerebrovascular diseases) of the Beijing Chaoyang Hospital, a tertiary hospital affiliated with Capital Medical University.

### Study design and sample

During a 2-month period in 2015, following a cross-sectional study design, a convenience sample of 250 patients with a diagnosis of chronic diseases (e.g., cardiovascular disease, cerebrovascular disease, respiratory disease, and diabetes mellitus) was surveyed via an anonymous questionnaire. Inclusion criteria were (1) had been diagnosed with one of the aforementioned chronic diseases for at least 3 months, (2) 18 years or age or older, (3) willing to participate, and (4) has ability for self-care. Exclusion criteria were (1) left questions unanswered on the questionnaire, (2) hospitalized with acute illness, (3) has a psychological or cognitive disorder, and (4) has a physical limitation that would inhibit physical activity.

### Instruments

Our self-designed, paper-and-pencil questionnaire (see Additional file 1) consisted of 24 questions, organized into three parts: (1) a demographic profile (i.e., five items) which comprised age, gender, marriage status, level of education, and work status; (2) patient health status (i.e., five items), which comprised height, weight, medical diagnoses, self-care status, and activity performance capacity; (3) current status of doing exercise, current status or using activity apps, and willingness of and barriers to using physical activity apps (i.e., 14 items).

For the assessment of the item that queried self-care status, which graded the response according to four levels, inter-rater reliability (i.e., *r* = 0.82) has been demonstrated by Su et al. [16]. Level 1 comprises patients who completely depend on others and do not perform self-care, which includes eating, bathing, and using the toilet. Level 2 comprises patients who are capable of self-care. Level 3 comprises patients who are not only capable of self-care, but also involved in social activities. Level 4 comprises patients who are engaged in full-time or part-time employment. The activity performance capacity was evaluated with the Karnofsky Performance Index [17], which features 11 response options that indicate functional status on a 0–100 point scale: 0 indicates *dead*, 10 indicates *dying*, and 100 indicates *normal, no complaints*. A score greater than 70 indicates a patient is able to “carry on normal activity” [18].

Current exercise status consisted of seven items (e.g., *How often do you exercise*). Current usage status consisted of three items (e.g., *Do you use a smartphone currently?*). Willingness and barriers to using physical activity apps included four items (e.g., *If there were a physical activity app designed for chronic disease patients like you, would you use it?*). We developed the questionnaire based on the review of related studies [19, 20]. To ensure validity, we pilot tested the questionnaire using two complementary approaches. First, the questionnaire was evaluated by five experts in nursing informatics, and we made modifications based on their suggestions. Second, we pilot tested the questionnaire with 20 patients. Based on their responses, we made minor adjustments to the questionnaire.

### Data collection procedure

One of the authors (CJ) was available when the questionnaire was distributed to the patients in the four mentioned outpatient departments during the 2-month period so that any of their questions could be answered. Explanations and clarifications were provided when necessary to ensure that respondents were clear about the aim of each question item. Responding to the questionnaire took each respondent approximately 15 min.

### Data analysis

Descriptive statistics were calculated for all items. Percentages and frequencies were evaluated for categorical variables, and means and standard deviation were determined for continuous variables. For all statistical tests, a *p*-value below 0.05 was considered statistically significant. A chi-squared test was conducted to analyze differences between (1) physical activity app users and non-users and (2) willingness to use apps and reluctance to use apps. SPSS version 16.0 was used to analyze the data.

### Ethical consent

This study was approved by the Capital Medical University Ethical Committee. A statement of consent was included with a brief description of the study in the introduction to the questionnaire, and the consent of participants was implied by the completion and return of the questionnaire, which was confidential and anonymous.

## Results

### Demographic and health characteristics

Questionnaires were completed by 218 of the 250 patients (i.e., 87.2% response rate) who were approached to take the survey. Demographic characteristics of the participants are summarized in Table 1. Participants were aged 20–69 (44.60 ± 9.44) years, their BMI was 17.21–31.25 (23.39 ± 2.65) kg/m^2^, and their Karnofsky Performance Index was 70–100 (73.39 ± 13.69). Additionally, 83.0% of the participants displayed a level 4 self-care ability; 17.0% displayed a level 3 self-care ability.

**Table 1:** Physical Activity App Usage by Chronic Disease Patients vis-à-vis Individual Factors

Individual Factors	Frequency n (%)	Physical Activity App Usage		χ^2^	*p*
		Yes (*n* = 76)	No (*n* = 142)		
Sex					
Male	85 (39.0)	28 (36.8)	5 (40.1)	0.226	0.634
Female	133 (61.0)	48 (63.2)	57 (59.9)		
Age (years)					
≤44	89 (40.8)	45 (59.2)	44 (31.0)	16.324	<0.001
>44	129 (69.2)	31 (40.8)	98 (69.0)		
Marriage status					
Married	179 (82.1)	58 (76.3)	12 (85.2)	2.667	0.102
Others	39 (17.9)	18 (23.7)	21 (14.8)		
Education					
Primary/secondary	73 (33.4)	19 (25.0)	54 (38.0)	3.773	0.052
Diploma/degree	145 (66.5)	57 (75.0)	88 (62.0)		
Employment status					
Yes	172 (78.9)	71 (93.4)	101 (71.1)	14.779	< 0.001
No	46 (21.1)	5 (6.6)	41 (28.9)		
BMI					
≤24	134 (61.5)	47 (61.8)	87 (61.3)	0.007	0.934
>24	84 (38.5)	29 (38.2)	55 (38.7)		
Medical diagnosis					
Cardiovascular	71 (32.6)	20 (28.2)	51 (78.1)		
Cerebrovascular	27 (12.4)	11 (40.7)	16 (59.3)	4.137	0.388
Respiratory	64 (29.4)	27 (42.2)	37 (57.8)		
DM	33 (15.1)	12 (36.4)	21 (63.6)		
More than one	23 (10.5)	6 (26.1)	17 (73.9)		
Exercise frequency					
None/seldom	48 (22.0)	8 (10.5)	40 (28.2)	10.311	0.016
Occasionally	63 (28.9)	22 (28.9)	41 (28.9)		
1–2 times/week	44 (20.2)	20 (26.3)	24 (16.9)		
3–4 times/week	63 (28.9)	26 (34.2)	37 (26.1)		
Exercise time					
<30 min	64 (29.4)	19 (25.0)	45 (31.7)	1.760	0.415
30–60 min	99 (45.4)	39 (51.3)	60 (42.3)		
>60 min	55 (25.2)	18 (23.7)	37 (26.1)		
Perceiving their chronic disease need doing reasonable exercises					
Yes	159 (72.9)	61 (80.3)	98 (69.0)	3.174	0.075
No	59 (27.1)	15 (19.7)	44 (31.0)		
Perceiving their chronic disease need exercise instructions					
Yes	113 (51.8)	46 (60.5)	67 (47.2)	3.530	0.060
No	105 (48.2)	30 (39.5)	75 (52.8)		
Need reminders to doing exercise					
Yes	78 (35.8)	26 (34.2)	52 (36.6)	0.768	0.420
No	140 (64.2)	50 (65.8)	90 (63.4)		
Need monitor physical status when doing exercise					
Yes	148 (67.9)	55 (72.4)	93 (65.5)	1.074	0.300
No	70 (32.1)	21 (27.6)	49 (32.1)		
Perceiving their chronic disease need professional support					
Yes	156 (71.6)	59 (77.6)	97 (68.3)	2.115	0.146
No	62 (28.4)	17 (22.4)	45 (31.7)		
Acceptance of charged apps					
Yes	26 (11.9)	7 (9.2)	19 (13.4)	0.819	0.025
No	192 (88.1)	69 (90.8)	123 (86.6)		

### Current exercise status and usage of smartphone apps

At the time of completing the survey, 92.7% of the respondents were using a smartphone, 34.9% had a history of physical activity app use, 18.8% were current users of a physical activity app, 65.1% had not used a physical activity app, and 72% knew about wearable devices. Table 1 shows the statistically significant differences among the demographic characteristics. Participants were divided into two groups according to whether or not they had used physical activity apps. Availability and use of mobile devices presents itself differently within different age groups (χ^2^ = 16.524, *p* ≤ 0.001); moreover, the difference between employment status and usage of physical activity apps was statistically significant (χ^2^ = 14.779, *p* ≤ 0.001).

### Willingness to use physical activity app for their health

53.7% of the participants were willing to use physical activity apps, 43.1% reported use of the app would depend on their condition, and only 3.2% were reluctant to use an app. Participants were divided into two groups according to their willingness to use physical activity apps (see Table 2). The willingness to use a physical activity app demonstrated statistically significant differences between age, education level, current smartphone user, having previous experience using physical activity apps, belief in the need to exercise, believing their disease required exercise instruction and support, belief in a need to monitor physical status when doing exercise, and an acceptance of a fee for apps (*p* < 0.05).

**Table 2:** The Willingness of Chronic Disease Patients to Use Physical Activity Apps vis-à-vis Individual Factors

Individual Factors	Willingness to Use Physical Activity Apps (n, %)		χ^2^	*p*
	Yes (*n* = 117)	No/Uncertain (*n* = 101)		
Sex				
Male (85)	41 (35.0)	44 (43.6)	1.65	0.198
Female (133)	76 (65.0)	57 (56.4)		
Age (years)				
≤44 (89)	60 (51.3)	29 (28.7)	11.42	0.001
>44 (129)	57 (48.7)	72 (71.3)		
Marriage status				
Married (179)	94 (80.3)	85 (84.2)	0.53	0.463
Others (39)	23 (19.7)	16 (15.8)		
Education				
Primary (73)	32 (27.4)	41 (40.6)	4.26	0.045
Diploma/degree (145)	85 (72.6)	60 (59.4)		
Employment status				
Yes (172)	98 (83.8)	74 (73.3)	3.58	0.058
No (46)	19 (16.2)	27 (26.7)		
BMI				
Standard (134)	76 (65.0)	58 (57.4)	1.29	0.255
Overweight (84)	41 (35.0)	43 (42.6)		
Medical Diagnosis				
Cardiovascular (71)	33 (46.5)	38 (53.5)		
Cerebrovascular (27)	15 (55.6)	12 (44.4)	2.62	0.624
Respiratory (64)	38 (59.4)	26 (40.6)		
DM (33)	19 (57.6)	14 (42.4)		
More than one (23)	12 (52.2)	11 (47.8)		
Exercise Frequency				
None/seldom (48)	21 (17.9)	27 (26.7)	9.92	0.019
Occasionally (63)	27 (23.1)	36 (35.6)		
1–2 times/week (44)	28 (23.9)	16 (15.8)		
3–4 times/week (63)	41 (35.0)	22 (34.9)		
Exercise time				
<30mins (64)	29 (24.8)	35 (34.7)	3.30	0.192
30–60mins (99)	54 (46.2)	45 (44.6)		
>60mins (55)	34 (29.1)	21 (20.8)		
Smartphone user				
Yes (202)	113 (96.6)	89 (88.1)	5.70	0.020
No (16)	4 (3.4)	12 (11.9)		
Had used physical activity apps				
Yes (76)	55 (47.0)	21 (20.8)	16.40	< 0.001
No (142)	62 (53.0)	80 (79.2)		
Perceiving their chronic disease need reasonable doing exercises				
Yes (159)	93 (79.5)	66 (65.3)	5.49	0.019
No (59)	24 (20.5)	35 (34.7)		
Perceiving their chronic disease need exercise instructions				
Yes (113)	75 (64.1)	38 (37.6)	15.22	< 0.001
No (105)	42 (35.9)	63 (62.4)		
Perceiving their chronic disease need professional support				
Yes (156)	96 (82.1)	60 (59.4)	16.60	< 0.001
No (62)	21 (17.9)	41 (40.6)		
Need encourage when doing exercise				
Yes (78)	43 (36.8)	35 (34.7)	0.78	0.429
No (140)	74 (63.2)	66 (65.3)		
Need monitor physical status when doing exercise				
Yes (148)	91 (61.5)	57 (56.4)	11.33	0.001
No (70)	26 (22.2)	44 (43.6)		
Acceptance of charged apps				
Yes (26)	19 (16.2)	7 (6.9)	4.47	0.038
No (192)	98 (83.8)	94 (93.1)		

### Barriers for users and perceived barriers for non-users

We also found that 46.0% (i.e., 35/76) of the physical activity users gave up on using physical activity apps. The reasons for this were (1) insufficient function (62.9%), extra cost (48.6%), security (42.9%), difficulty to use (40.0%), and extra data cost (37.1%). The barriers and perceived barriers of chronic disease patients to using physical activity apps see Table 3.

**Table 3:** The Barriers and/or Perceived Barriers of Chronic Disease Patients to Using Physical Activity Apps

Survey Item	Total (n, %) 218	Physical Activity App Usage (n, %)		χ^2^	*p*
		Yes (*n* = 76)	No (*n* = 142)		
1. Functions do not meet needs (i.e., function is insufficient).	107 (49.1)	47 (61.8)	60 (42.3)	7.601	0.006
2. It is not easy to use (i.e., difficulty to use).	103 (47.2)	29 (38.2)	74 (52.1)	3.868	0.049
3. There is an extra fee to use the app (i.e., extra cost).	91 (41.7)	31 (40.8)	60 (42.3)	0.044	0.835
4. Worried about personal information disclosure (i.e., security).	81 (37.2)	31 (40.8)	50 (35.2)	0.660	0.252
5. Using apps will use more mobile plan data (i.e., extra data cost).	58 (26.6)	27 (35.5)	31 (21.8)	4.755	0.029

## Discussion

### Current usage

The majority (92.7%) of participants owned a smartphone, which may be explained because our survey was conducted in the capital city and economic center of China. Nielsen estimates that the rate of smartphone penetration in China is increasing exponentially, especially in urban areas, which exhibited a rise to over 90% penetration in 2015 [21]. Because our participants were representative of the typical patient population in a tertiary care hospital in urban Beijing, which is similar to the patient population in other major Chinese cities in China (i.e., patients from suburbs typically seek care in the central urban areas of the cities), we can assume that smartphone ownership in large cities across the county parallels the ownership trends in Beijing.

Few studies—in either China or the West—specifically have examined the use of smartphone physical activity apps. Our results reveal a statistically significance difference between usage of physical activity apps and age (χ^2^ = 16.524, *p* < 0.001) as well as employment status (χ^2^ = 14.779, *p* < 0.001). The data presented in Table 1 indicate that patients under 44 years old who are still working (full- or part-time) are likely to use physical activity apps. Similarly, Davies et al. found that younger participants (i.e., < 44 years of age) are positive to use apps compared to the older participants and explained that younger individuals are accustomed to mobile technology and more reliant on it for conducting daily activities [22]. To fully realize the potential of mobile technologies in a health care context, the needs of the middle-aged and the elderly need to be carefully addressed in all strategies relating to mobile technology in a healthcare context.

### Willingness to use

More than half (53.7%) of our participants reported their willingness to utilize physical activity apps designed for chronic disease patients. This suggests that Chinese health educators could promote such apps to chronic disease patients to not only encourage them to exercise, but also enable them to receive and share useful information, which could further promote self-management of physical activities. This mirrors the results of similar studies in Western countries. For example, Winter et al. argues that the evidence base is sufficiently promising to encourage the patients who want to use mobile technologies and cellphone apps to increase their physical activity [23], and Vandelanotte et al. show that this is the case in Australia [24].

Another statistically significant (*p* < 0.05) trend that emerged was that individuals more likely to use physical activity apps are younger (i.e., < 44 years of age), more educated, current smartphone users, have used physical activity apps, and can accept paying for apps; moreover, they believe that (1) they need to exercise, (2) their disease requires exercise instruction and support, and (3) they need monitor their physical status when doing exercise. Past studies (e.g., Davies et al. and Lee et al.) also have demonstrated that, compared to the elderly (i.e., 65 years of age), younger (i.e., < 44 years of age) individuals demonstrate a higher likelihood of using mHealth apps and technology [22, 25]. Although a growing number of mHealth apps currently are being made available to support the patients in both China and Western countries, such apps may not be designed to account for age-specific requirements.

Our participants reported being more willing to use apps promoted by healthcare professionals, which is echoed in the literature. For example, Boudreaux et al. [26] assert that mHealth apps developed by healthcare organizations boast higher patient ratings and higher patient comfort scores [26], and Visser et al. recommends that medical professionals should be involved during all stages of app development [27]. Through a survey of middle-aged male patients, Illiger et al. discovered that the majority considered intervention technology more acceptable if doctors use the devices to illustrate something (e.g., using the device for patient education and providing or illustrating information) [19], Moreover, Davies et al. report that the general population is willing to receive training on using apps from pharmacists for medicine adherence [22], Although these studies report primarily on medicine adherence mobile apps usage, they do shed light on the use of physical activity apps.

Patients benefit from the involvement of medical professionals; however, healthcare researchers in Western contexts are concerned about how current smartphone applications tend to lack medical professional involvement and peer review [28], and the same situation is occurring in China. For example, Xiao et al. [29] reveals that there is little evidence of the involvement of health professional in the formation of the CVD-related apps available in China. Concerns such as these combined with our results may provide recommendation strategies for the future oversight and design of physical activity apps for Chinese patients.

Moreover, concerns about using mobile technologies in a medical context are often associated with whether or not patients are familiar with using mobile devices, which is influenced by access to such a device [19], Therefore, widespread ownership of smartphones and use of apps plays a role in the use of this technology in health care. Moreover, we found that the willingness to use a physical activity app was associated with increased education, which suggests that more education allows for an easier acceptance of new ways of doing things. For example, Krebs et al. [20], demonstrate that, in the United States, having downloaded a health app is associated with not only having greater than an high school education, but also younger age and higher income; moreover, other demographics (e.g., ethnicity and gender) are contributors [30]. Furthermore, the suggestions given to patients by healthcare professionals on using such technology also may contribute to the patients’ proclivity of using the apps [28].

### Barriers for users and perceived barriers for non-users

In our study, insufficient function, security, and extra cost were the top barriers to using physical activity apps among users, while difficulty to use, insufficient function, and extra cost were the top perceived barriers among non-users. That 61.8% of the physical activity app users considered insufficient function the top barriers reveals that the physical activity apps currently available may not satisfy the needs of chronic disease patients. Performing content analysis about available physical activity apps, King et al. found that, despite an explosion of cellphone apps targeting physical activity and health behaviors, few of the apps are based on theoretical constructs and empirical evidence; therefore, apps must take measurable parameters into consideration such as quantified goal-setting, behavioral feedback, and problem solving around barriers to behavioral change [31]. In addition, in a similar study examining user preferences of physical activity apps, Rabin and Bock recommend integrating the following features into health-related apps: automatic tracking, progress toward physical activity goals, music features, incorporates several types of exercise, well-documented features and user-friendly interfaces. Chronic disease patients may have specific needs concerning app functionality comparing to general users, which suggests that subsequent research could target the design of physical activity apps to particular users (e.g., cardiovascular disease patients) [32].

Another interesting finding was that although 47.2% of our participants reported difficulty of use as one of main barriers to using physical activity apps, more non-app users were concerned about this than app users (χ^2^ = 3.868, *p* = 0.049). Similarly, Illiger et al. report that clinical staff worry that devices might be too complicated for patients to use in a health context [19]. Perhaps current physical activity apps are easier to use than non-app users think, which is something health educators who are interested in using apps to promote physical activity among chronic disease patients can consider.

Our results indicate that one of the major issues with physical activity apps in the Chinese context is the concern over data security, which also is an issue in the Western context. For example, Boulos et al. argue that a fundamental issue with mHealth apps is not only their ability to collect and transmit personal information including health status, but also who has access to this data [33]. This is demonstrated in Illiger et al. [19] who report that their patients worried about data protection when physicians using mobile devices—roughly every fifth participant did not want his or her doctors to save or process their individual health related data on a mobile device. In light of these concerns, some researchers have integrated information quality evaluation scales, such as the Silberg scale and the MARS scale, into their evaluation of the usefulness and information quality of the applications in question [29, 34, 35]. Moreover, other researchers have highlighted the paucity of regulation and data security issues surrounding patient apps [28, 29], which begs the question about the role governmental bodies could play in regulating health apps to ensure the security of the associated patient data.

Our participants also reported that being charged extra fees for using apps was a concern, and this concern was larger for current app users than non-users (χ^2^ = 4.755, *p* = 0.029). In the Western context, Krebs and Duncan reported that nearly half of their app users stopped using some health apps because of high data use and other hidden costs [20]. These practical barriers to using health apps reveal several concerns that need to be addressed in subsequent study in both China and Western countries.

Our findings raise a number of clinical implications for patients living with chronic diseases. Mobile health realized via smartphones and their apps is a new technological modality that empowers individuals to manage their chronic diseases, facilitates rehabilitation programs, assists clinicians with making medical diagnoses, facilitates outreach efforts in developing countries [35], all of which can make the utilization of healthcare resources more efficient. Our data reveal that Chinese patients comprise a potentially huge user cohort for mHealth via smartphone applications, as not only the number of users of mobile devices and wireless, but also the number of patients with chronic diseases rapidly increases. Moreover, our survey demonstrates that Chinese patients possess similar concerns with Western patients about using smartphone applications (i.e., safety), which run in parallel with the growing concerns of healthcare workers, health promoters, and practitioners in related disciplines about the design, clinical suitability, and information quality of these apps. Furthermore, as a result of regulatory policies and language differences, Chinese patients are unable to access particular applications that are commonly accessible in Western countries, and this should alert health researchers and healthcare worker to place more effort in creating and introducing more effective mHealth applications for smartphones in the Chinese healthcare context.

## Conclusion

Physical inactivity is not only a common risk factor for most of chronic diseases (e.g., cardiovascular disease, diabetes, and cancer), but also becoming a significant public health concern worldwide. To minimize this risk factor, physical activity interventions are being developed and disseminated among patients. Smartphone apps are promising vehicles for delivering physical activity interventions. The results of this study, one of the first to evaluated the current use, willingness to use, and perceived barriers to using physical activity apps among Chinese chronic disease patients, provide some insight into the opinions of these patients vis-à-vis the design and function of physical activity apps. Our results suggest that the acceptance and uptake of physical activity apps is dependent upon a constellation of factors concerning not only the patient (e.g., age and education level) but also the app and the intervention (e.g., appropriate promotion, education strategies, and functions). Our findings, and the scarcity of literature on the matter, emphasize the need for further research concerning the use of mobile devices in medical settings, in both China and Western countries, to fully realize the potential that mobile technologies can offer medicine, while respecting the needs and concerns of the users. Moreover, our findings have implications for healthcare professionals working to improving the management of physical activity and chronic disease management in China and elsewhere. Subsequent study can target not only apps designed for a specific intervention or area of application, but also what makes these apps attractive for potential patients or prevents potential users from using them.

## Strengths and limitations

The study has several limitations. The first is its limited participant sample drawn from a single tertiary hospital in Beijing. Because the number of users of smartphones in metropolitan areas in China such as Being is larger than the number in outlying suburban and rural areas, our Beijing-based participant sample constrains the generalizability of our results. The second is a methodological limitation: we recruited participants relative to four chronic diseases, and the needs of these patients—along with their barriers to physical activity—likely differ across the four diseases. Therefore, our results only provide a general picture of chronic disease patients in China, and subsequent studies could investigate patients suffering a single chronic disease to elucidate the specific needs and barriers associated with these patients vis-à-vis smartphone health apps. The third limitation results from our cross-sectional data. Although data of this sort is helpful in examining the physical activity app usage of our participants at one point in time, it cannot account for how patients are likely to change their use patterns overtime, which points to the need for a subsequent longitudinal study. Last but not least, a self-reported survey was used, which raises questions about the reliability of the instrument and the data collected. For example, our survey instrument lacked open-ended items that could gain more specific information from respondents about, like why they perceived the function of current physical activity apps to be insufficient. Nevertheless, the strength of this study is that it is among the first to investigate the potential of physical activity app to promote a healthy lifestyle among Chinese chronic disease patients. As such, it provides useful information for recognizing the facilitators and barriers to the use of smartphone apps among this patient population, which can lay the groundwork for the future conceptualization of new applications.

## Additional file

Additional file 1: Survey of Physical Activity App Use Among Chronic Disease Patients (English Version). The questionnaire describes the participants’ demographic profile, patient health status and current status of doing exercise, current status of using activity apps, and willingness of and barriers to using physical activity apps. (PDF 138 kb)

## Abbreviations

App: Application
CVD: Coronary heart disease
mHealth: Mobile health

## References

[1] Information Office of the State Council (2014). The PRC medical and health services in China.

[2] Glynn LG, Hayes PS, Casey M, Glynn F, Alvarez-lglesias A, Newell J, OLaighin G, Heaney D, O’Donnell M, Murphy AW (2014). Effectiveness of a smartphone application to promote physical activity in primary care: The SMART MOVE randomised controlled trial. Br J Gen Pract.

[3] Cleland CL, Tully MA, Kee F, Cupples ME (2012). The effectiveness of physical activity interventions in socio-economically disadvantaged communities: A systematic review. Prev Med.

[4] Dallinga JM, Mennes M, Alpay L, Bijwaard H, Baart de la Faille-Deutekom M (2015). App use, physical activity and healthy lifestyle: a cross sectional study. BMC Public Health.

[5] Casey M, Hayes PS, Glynn F, OLaighin G, Heaney D, Murphy AW, Glynn LG (2014). Patients’ experiences of using a smartphone application to increase physical activity: the SMART MOVE qualitative study in primary care. Br J Gen Pract.

[6] Masse L, Direito A, Knight E, Stuckey MI, Prapavessis H, Petrella RJ (2015). Public health guidelines for physical activity: is there an App for that? a review of android and apple app stores. JMIR Mhealth Uhealth.

[7] Liu C, Wang Q, Qian J (2015). The influence of sports apps on the promotion of physical exercise behavior and the formation of exercise habits. J Nanjing Sport Inst.

[8] China Internet Network Information Center(CNNIC) (2015). The 36th Chinese internet network development statistic report.

[9] Dolan B (2011). Report: 13K iPhone consumer health apps in 2012 | mobihealthnews. mobihealthnews.com.

[10] Middelweerd A, Mollee JS, van der Wal CN, Brug J, Te Velde SJ (2014). Apps to promote physical activity among adults: a review and content analysis. Int J Behav Nutr Phys Act.

[11] Wang YL, Sun L, Xu YH, Xiao Q, Chang P, Wu Y (2015). Comparison and analysis of top 10 exercise android apps in mainland China. Stud Health Technol Inform.

[12] Fanning J, Muller S, Mullen SP, Mcauley E (2012). Increasing physical activity with mobile devices: a meta-analysis. J Med Internet Res.

[13] Stephens J, Allen J (2013). Mobile phone interventions to increase physical activity and reduce weight: a systematic review. J Cardiovasc Nurs.

[14] Coughlin SS, Whitehead M, Sheats JQ, Mastromonico J, Smith S (2016). A review of smartphone applications for promoting physical activity. Jacobs journal of community medicine.

[15] Schoeppe S, Alley S, Van Lippevelde W, Bray NA, Williams SL, Duncan MJ, Vandelanotte C (2016). Efficacy of interventions that use apps to improve diet, physical activity and sedentary behaviour: a systematic review. Int J Behav Nutr Phys Act.

[16] Su CY, Lu XH, Chen W, Wang T (2009). Promoting self-management improves the health status of patients having peritoneal dialysis. J Adv Nurs.

[17] Karnofsky DA, Burchenal JH, MacLeod CM (1949). The clinical evaluation of chemotherapeutic agents in cancer. Evaluation of chemotherapeutic agents.

[18] Parkerson GR Jr,Gutman RA.Predictors of functional health status of end stage renal disease patients. Health Care Financ Rev. 1997 Summer;18(4):37–49. PMC4194472 10175612

[19] Illiger K, Hupka M, von Jan U, Wichelhaus D, Albrecht U-V. Mobile Technologies: Expectancy, Usage, and Acceptance of Clinical Staff and Patients at a University Medical Center. Eysenbach G, ed. JMIR mHealth and uHealth. 2014;2(4):e42. doi:10.2196/mhealth.3799. 10.2196/mhealth.3799 PMC4259908 25338094

[20] Krebs P, Duncan DT (2015). Health app use among US mobile phone owners: a national survey. JMIR mHealth uHealth.

[21] Nielsen. Chinese Smartphone Market Now Driven by Upgrading. 2015 Available at http://www.nielsen.com/cn/en/press-room/2015/Nielsen-Chinese-Smartphone-Market-Now-Driven-by-Upgrading-EN.html. Accessed 8 Feb 2017.

[22] Davies MJ, Kotadia A, Mughal H, Hannan A, Alqarni H (2015). The attitudes of pharmacists, students and the general public on mHealth applications for medication adherence. Pharm Pract (Granada).

[23] Johnston W, Hoffman S, Thornton L (2014). Mobile health: a synopsis and comment on ‘increasing physical activity with mobile devices: a meta-analysis’. Translational Behavioral Medicine.

[24] Vandelanotte C, Caperchione CM, Ellison M, George ES, Maeder A, Kolt GS, Duncan MJ, Karunanithi M, Noakes M, Hooker C, Viljoen P, Mummery WK (2013). What kinds of website and mobile phone-delivered physical activity and nutrition interventions do middle-aged men want?. J Health Commun.

[25] Lee JA, Nguyen AL, Berg J, Amin A, Bachman M, Guo YQ, Evangelista L (2014). Attitudes and preferences on the use of mobile health technology and health games for self-management: interviews with older adults on anticoagulation therapy. JMIR mHealth and uHealth.

[26] Boudreaux ED, Waring ME, Hayes RB, Sadasivam RS, Mullen S (2014). Evaluating and selecting mobile health apps: strategies for healthcare providers and healthcare organizations. Translational Behavioral Medicine.

[27] Visser BJ, Buijink AWG (2012). Need to peer-review medical applications for smart phones. J telemed and telecare.

[28] Zhang MWB, Ho RCM, Hawa R, Sockalingam S (2016). Analysis of the information quality of bariatric surgery smartphone applications using the silberg scale. Obes Surg.

[29] Xiao Q, Lu S, Wang Y, Sun L, Wu Y. Current Status of Cardiovascular Disease-Related Smartphone Apps Downloadable in China. Telemed J E Health. 2017;23(3):219–225. doi: 10.1089/tmj.2016.0083. Epub 2016 Jun 29. 10.1089/tmj.2016.0083 27356156

[30] Smith A. The Smartphone Difference. Pew Research Center Washington, DC:Pew Internet & American Life Project; 2015. Available at http://www.pewinternet.org/2015/04/01/us-smartphone-use-in-2015/. Accessed 19 Sept 2016

[31] King AC, Hekler EB, Grieco LA, Winter SJ, Sheats JL, Buman MP, Banerjee B, Robinson TN, Cirimele J (2013). Harnessing different motivational frames via mobile phones to promote daily physical activity and reduce sedentary behavior in aging adults. PLoS One.

[32] Rabin C, Bock B (2011). Desired features of smartphone applications promoting physical activity. Telemed and e-Health.

[33] Boulos MNK, Brewer AC, Karimkhani C, Buller DB, Dellavalle RP (2014). Mobile medical and health apps: state of the art, concerns, regulatory control and certification. Online Journal of Public Health Informatics.

[34] Mani M, Kavanagh DJ, Hides L, Stoyanov SR (2015). Review and evaluation of mindfulness-based iPhone apps. JMIR mHealth and uHealth.

[35] Zhang MWB, Ho RCM (2015). Enabling psychiatrists to explore the full potential of E-health. Front Psychiatry.

