# Supplementary material for: Facilitators and barriers to using physical activity smartphone apps among Chinese patients with chronic diseases
Source: BMC Med Inform Decis Mak. 2017 Apr 19;17:44. doi: 10.1186/s12911-017-0446-0 (PMC5395907; doi:10.1186/s12911-017-0446-0)
Supplement: Additional file 1: — Survey of Physical Activity App Use Among Chronic Disease Patients (English Version). The questionnaire describes the participants’ demographic profile, patient health status and current status of doing exercise, current status of using activity apps, and willingness of and barriers to using physical activity apps. (PDF 138 kb) [file 12911_2017_446_MOESM1_ESM.pdf]

No：□□□ Date:______Month______Day

Survey of Physical Activity App Use Among Chronic Disease Patients (English Version)
You are invited to participate in this survey. The survey is designed to understand your current status and willingness of using smartphone physical activity applications. It will take approximately 15 minutes to complete. The completion of survey is voluntary and anonymous. Consent is implied by survey completion and submission. Your participation will be greatly appreciated as it will contribute to an improvement of patients physical activity self-management.<br>To indicate your answer please CIRCLE the number alongside the answer that comes closest to your opinion or wr ite your answer in the space provided.
Q1. Age: _________
Q2. Gender: 1. Female 2. Male
Q3. You are currently (circle only one)<br>1. Single 2. Married 3. Separated 4. Widowed 5. Divorced 6. Other
Q4. Please circle your final level of education：<br>1. Primary or under 2. Secondary 3. Diploma 4. Degree or higher
Q5. What is your current work status? (circle only one):<br>1. Full-time work 2. Part-time work 3. Not working
Q6. Your height _________cm
Q7. Your current weight _________ kg
Q8. According to your medical diagnosis (at least 3 months), please indicate which chronic conditions you have:<br>1. Cardiovascular disease 2. Cerebrovascular disease<br>2. Respiratory disease 4. Diabetes mellitus
Q9. Which of the following conform to your current self-care status?<br>1.You are completely depend on others and do not perform self-care, which includes eating, bathing, and using the toilet.<br>2.You are capable of self-care.<br>3.You are not only capable of self-care, but also involved in social activities.<br>4.You are engaged in full-time or part-time employment.
Q10. Please circle the number which reflect your activity performance?<br>0 indicates dead, 10 indicates dying, and 100 indicates normal—no complaints.<br>0 10 20 30 40 50 60 70 80 90 100
Q11. How often do you exercise?<br>1. Never/seldom 2. Occasionally 3. 1–2 times/week 4. 3–4 times/week
Q12. How long do you do exercise each time?<br>1. < 30 min 2. 30–60 min 3. > 60 min
Q13. Do you need to be reminded by others to exercise?<br>1. Yes 2. No
Q14. Do you think your disease need reasonable exercise?<br>1. Yes 2. No
Q15. Do you think your disease need exercise instruction?

1. Yes 2. No
Q16. Do you think you need the suggestions of professionals (doctors or nurses) on doing exercise?<br>1. Yes 2. No
Q17. Do you think you need monitor your physical status, such as heart rate, when exercising?<br>1. Yes 2. No
Q18. Do you currently use a smartphone currently?<br>1. Yes 2. No
Q19. Have you used a physical activity smartphone application? Smartphones are handheld computers that can run application software, so-called "apps" that can be download from so-called "App Stores" available on the Internet.<br>1. Yes 2. No
Q20. Have you heard of wearing physical activity devices, such as smart bracelets?<br>1. Yes 2. No
Q21. If there were a physical activity app designed for chronic disease patients like you, would you use it?<br>1. Yes 2. Uncertain, it depends 3. No
Q22. If there were a physical activity app for your disease that you had to pay for, would you use it?<br>1. Yes 2. No
Q23. Do you think you need monitor your physical status when you doing exercise?<br>1. Yes 2. No
Q24. Which of the following factors influenced you to use a physical activity app?<br>Functions do not meet needs (i.e., function is insufficient).<br>1. It is not easy to use (i.e., difficulty to use).<br>2. There is an extra fee to use the app (i.e., extra cost).<br>3. Worried about personal information disclosure (i.e., security).<br>4. Using apps will use more mobile plan data (i.e., extra data cost)
